# PROD-ALERT: Psychiatric restraint open data—analysis using logarithmic estimates on reporting trends

**DOI:** 10.3389/fdgth.2022.945635

**Published:** 2022-08-12

**Authors:** Keith Reid, Owen Price

**Affiliations:** ^1^ Positive and Safe Care, Cumbria, Northumberland Tyne and Wear NHS Foundation Trust, Newcastle upon Tyne, United Kingdom; ^2^ Positive and Safe Care, Northumbria University, Newcastle upon Tyne, North East England, United Kingdom; ^3^ Division of Nursing, Midwifery & Social Work, The University of Manchester, Manchester, England, United Kingdom

**Keywords:** human rights, law, restrictive practice, statistical methodology, open data

## Abstract

**Aims and Methods:**

Restraint reporting varies, which undermines regulation, obfuscates analyses, and incentivises minimisation. The English Mental Health Units Use of Force Act 2018, “Seni's Law” mandates reporting. This paper analysed open data from all psychiatric and learning disability institutions in England from September 2020 to August 2021. We correlated logarithms of “people restrained per month”, against “bed days” per month and “people under legal mental health detention” per month, per institution. We designated institutions reporting some restraint for at least 11 of 12 months as reporting “completely” and used their trend to infer rates from non-“complete” institutions. Allowance was made for size. Our a priori manual can be shared on request.

**Results:**

Logarithms of people restrained per month and bed-days per month correlated among complete reporters: R^2^ 0.90 (2.s.f). Persons detained per month also correlated with restraint: R^2^ 0.78. “Partial” institutions reported intermittently. “Joiner” institutions reported firstly null, then substantive reporting. “Null” institutions (including the largest) reported no restraint. Precisely-reporting institutions with high inverse variance between months reported similar restraint-rates but less-precise reported lower rates. In institutions reporting no restraint, two independent “true rate” estimations, by bed-days or people detained, correlated across institutions: R^2^ 0.95. Inference from size suggested non-complete reporters restrained 1,774 people in England per month 95% CI (1,449–2,174).

**Clinical implications:**

Restraint remains under-reported. Institutional size explains most restraint variation among complete reporting institutions, 90% of R^2^. Institutional restraint reports can be compared per-bed per-month. Rates of people detained are a useful independent “checking” comparator in England.

## Introduction

Seni's law, the Mental Health Units Use of Force Act 2018, requires institutions in England and Wales to report restraint (1). In 2013, a Freedom of Information (FOI) request regarding restraints elicited incomplete responses, and led to high levels of publicity and concern (2). That report acknowledged the incompleteness, and that it presented figures unadjusted for size and need.

Variable reporting is well attested to in international literature. Steinert et al. 2010 in their systematic review of data on the use of seclusion and restraint called it “scarcely available”. The intent was description of restraint per person in geographical areas per year (3). Janssen et al. 2011 in their review of Dutch restraint figures, further considered the difficulties of restraint data, standardised reporting of incidents, and framed cogent levels of analysis as geographical and institutional (4). In those terms ours is an institutional level of analysis using metrics chosen by a state.

Accurate capture/reporting of restraint data maintains human rights, improves metrics, and can map social determinants of restraint. Insightful and candid institutions can assess how similar they are, how well they are performing and reporting. Without accurate reporting or capturing of data on people restrained there are perverse incentives to minimise, or even deny that restraint occurs.

NHS Digital publishes a set of minimum mental health metrics, the “MHMDS” (5). Data for September 2020 to August 2021, self-reported monthly by institutions, was available in November 2021. These are open data, which we will share our clean version of on request, along with our manual for analysis. They are highly complex and detailed data, but *inter alia*, they include the following governmentally-required monthly metrics.

MHS24 “Bed Days” per month is a measure of size. MHS09 is an absolute count of how many people were involuntarily detained at any point for any length of time during the reporting period including continued detention of people detained. This paper calls it “people detained”. MHS76 is a monthly measure of how many people were subject to at least one restraint in that institution in that reporting month including people restrained in previous months. This paper calls it “people restrained”. Some institutions reported restraint at least 11 months a year and were assigned “complete” reporters. Other reporting styles are categorised below in simple terms.

People detained and people restrained might be expected to correlate because detention should be considered following restraint. People detained is a safe comparator because it is independently scrutinised, unambiguous, and people restrained is robust to negotiable restraint definitions. Each makes sense in terms of the other, and can be easily verified, by regulators, with patients.

The three comparisons were chosen to give independence between means of acquisition. Restraints are counted by safety systems. Legal administrators count people detained and are checked by regulators. Counting bed-days does not rely on the other two counts and is a basic task. The repeated measures given by these three monthly reports allow consideration of variance.

This paper aimed to address uncertainty regarding true figures of English mental health restraint, using only public data, funnel plots and simple trends. This was done prior to Seni's law's enforcement of central reporting – one optimistic view may be that we took a baseline. Then, having performed complete funnel plotting and scatter graphing and inference calculations, following a pause of three months during peer review, as a secondary follow-up, we used bed days to predict the December restraint figures for the only two null-reporting institutions who had converted to reporting.

## Methods

No ethical permissions were needed for public data. Analysis, including the manual, is intended to be shared freely on any request. Excel was used for reproducibility and coproduction with patients and the public. A manual settled methods prior to receipt of last data. Methods were logged with stakeholders a priori. Four institutions were excluded. These four exclusions comprised two institutions which closed; plus two children's hospitals which had no people detained. This left 84, which was all hospital institutions in the data set.

NHS Digital round their figures. Reports of [“26, 21, 11, 6, 1, 0, missing”] would be rounded to: [“25, 20, 10, 5, *, *, *”]. The asterisk * can be taken to mean ≤5. For the rest of the current paper “null” signifies “*”.

Institutions were categorised. “Small” were the continuous series of smallest institutions with “null” reports commensurate with size and having other monthly reports around 5 and 10. “Complete” institutions were bigger than “small” with one or less nulls. To interpolate single nulls among complete reporters, the last observation was carried forward, else next carried back (for month 1), both within the 12 months. Other institutions were neither small nor complete, and sub-categorised as “*Partial*”, “*Joiner*”, or “*Null*”.

By definition, “Joiners” consistently reported null then consistently reported restrained people – reporting none then many. “Partial” reporter institutions had intermittent null but were not small enough to be in the continuous “small” series - reporting many people restrained then none then many, etc. “Null” report institutions reported only null all year despite not being small – reporting none throughout while being the size of trusts reporting many. Small reporters with null restraints, and “joiner” or “partial” Bed-Day reporting style were categorised “null” – if bed counts were partial we did not trust restraint counts.

Data downloaded early in real time showed a log/log trend so log-relationships were settled, as the field of analysis, before receipt of final data. Base 2 was chosen, for logs, for compatibility with planned statistical tests.

**Figure 1:**
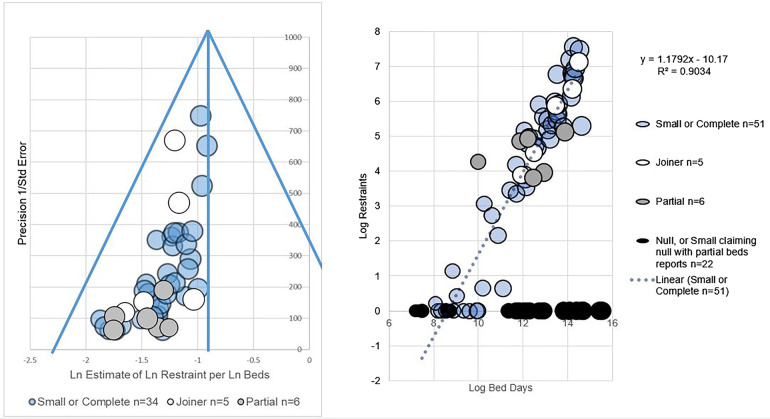
(**A**) shows a funnel plot of LnRestraint/LnBeds. (**B**) shows an L-sign scatter plot of LnRestraint/LnBeds.

It is convenient to discuss base 2 logarithms. Consider an institution with 500 beds and 65 restrained persons in a month after rounding, which is 14,000 bed days per month, in April. 2^2^ (2_i_*2_ii_) is 4. 2^7^ (2_i_*2_ii_*2_iii_…2_vii_) is 128. Log_2_ 14,000 gives LnBeds 13.77 because 2^13.77^∼14,000. The 65 restrained people gives LnRestraint 6.02 because 2^6.02^∼65. The Ln/Ln ratio between LnRestraint people and LnBeds would be 6.02/13.77. Graphical plotting show the pattern as in Figure 1B.

Each institution's report estimates a relationship between e.g., size and restraint in log/log graph terms. Each is an estimate of “slope”, which trend can be firstly expressed as y = mx + c the line equation and secondly give a measure of variance. Variance allows confidence intervals. For log people restrained per log bed days, such estimates of slope appeared normally distributed on visual inspection, so confidence intervals of slope could be estimated.

R^2^, coefficient of determination, assessed correlations between log metrics. Precision was 1/Standard Error in funnel plots, which is the convention. Laplace correction of 1 was applied to all data to allow log of null to be 0.

## Results

General patterns of reporting were as follows. The set of institutions comprised 84. That is all institutions in England who reported mental health people detained, reported mental health bed days, or reported restraint (the spirit of legislation is that they should report all three), and who were not one of the four excluded institutions. The biggest “small” institution for people restrained had around 2200 monthly bed-days. The biggest “small” institution people detained had around 1,000 bed-days because detention is more common than restraint. Institutions of these size and smaller could not be impugned when they reported null restraint, because they also had months with 5 or 10 persons restrained, as did similar sized institutions.

Regarding variance in reporting, see funnel plot of Figure 1A which funnel-plots estimates of LnRestraint per LnBeds. Both funnel plots showed more precisely-reporting institutions giving similar, higher estimates of the ratio of LnRestraint to either independently measured comparator (LnBeds or LnPeople detained). The right hand sides of funnels were sparsely filled.

*n* = 41 small or complete restraints per size estimates could be calculated. *n* = 33 small or complete estimates of restraints per people detained could be calculated, 5 partial, 4 joiners. 33 and 41 are not additive; they are separate analyses. In other words, half of institutions could not even be included in either funnel plot.

On visual inspection, partial reporters had less precision (inverse variance) than joiners in both types of graph, namely funnels and scatters. That is to say the white circles are higher up the funnel plot Figure 1A and closer to the trend line than the grey circles Figure 1B.

Regarding correlations of size and restraint, in its scatter plot, correlation of “small or complete” institution LnRestraint per LnBeds from complete reporters had R^2^ of 0.90 (2sf), see Figure 1B. That is to say variance in LnBeds largely explains variance in LnRestraint where we have full data.

Joiner reporters did not contribute to calculation of the slope of the LnRestraint per LnBeds trend but fell close to it (included in white for visual comparison).

Null reports contribute the horizontal limb of an “L” on the scatter (black) and, as stated above, are absent from funnels, having no calculable precision. Formally, a report of 0,0,0,0…, or a single report, has no variance from which to calculate “precision”, even if it were “true”, so null reporters were not funnel plotted.

Slope estimations for LnRestraints/LnBeds approximated normal distribution on visual inspection. This allowed an average and confidence limits for estimates of monthly person restrained derived from trends in institution size and person restrained. The sum of average estimates by size of people per month restrained in institutions with non-“complete” restraint reporting style estimated was 1,774 95% CI (1,449–2,174), per month. We suggest this can be extrapolated to 12*1,774 for the year, with caution.

**Figure 2:**
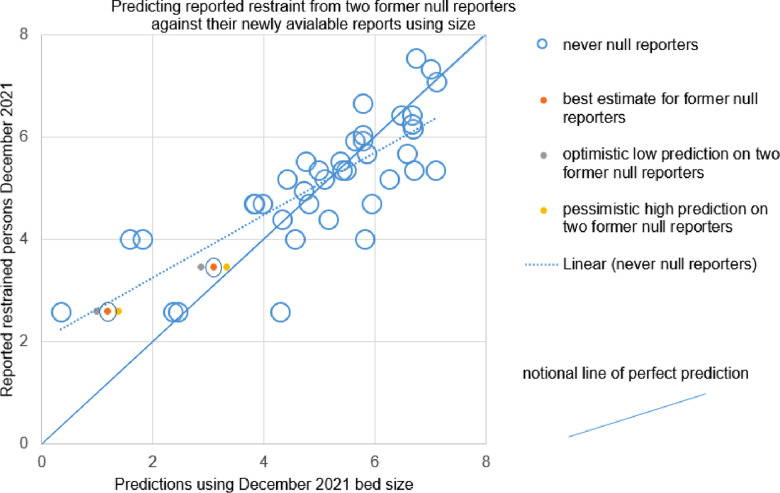
Shows pessimistic, optimistic and best interpolations based on 95% confidence intervals for two providers which moved from null reporting through the year of interest, to reporting some restraint by December. The dashed line is the trend amongst reporters which were never null. The complete line can be seen to be a notional line representing perfect prediction. The two “new joiner” providers sit between the trend and perfect prediction.

Similarly the 2 late joiner trusts fell near the trend for complete reporter LnRestraint per LnBeds, see 95% confidence interval predictions in Figure 2. This is a weak finding but could have disproven the trend.

The most striking result requiring little quantitative detail is that very largest institutions report null.

Regarding correlations of restraint and detention, LnRestraint/LnDetentions showed R^2^ of 0.78 (2 sf) for small or complete reporters and had an equivocal distribution of slope estimates.

A priori we compared detention and size-based estimates of restraint. LnDetention-based and LnBeds-based estimates of restraint correlated very highly R^2^ = 0.95 (6) in estimating restraints from null reporters, barring one institution apparently (post hoc) criticised by CQC regarding MHA reporting.

The two independent estimates agreed in absolute terms for null institutions with estimated restraints less than 50 per month. They diverged (while still correlating at 0.95 overall) for larger institutions. Under that divergence, LnBeds always provided a higher estimate of restraint than LnDetention: e.g. 279 vs. 61 restraints, for the largest “null”, predicted using LnBeds and LnDetention respectively. In other words, number of people detained per month and number of people restrained per month make correlated predictions. The true rate remains unknown in null reporters, by definition.

Then, following a pause of three months during peer review, we opportunistically used bed days to predict the December 2021 restraint figures for the only two null reporting institutions who became Joiner reporters. Their 95% confidence intervals lay between a notional line of perfect prediction and the actual trend between prediction by size and reported figures, see Figure 2. That is to say, if we had been exactly right they would have been on the diagonal down the middle, continuous line in Figure 2. If they had been exactly on trend they would have been on the dashed line representing the trend among complete reporters, Figure 2. For both institutions, the 95% confidence intervals lay between the dashed and complete line. Two estimates is not a large enough number to give a meaningful summary of the “success” of two estimates. The current paper merely points out that the institutions a) did not have no restraint as claimed through the original year b) had visual face validity among the trend on visual inspection with complete reporters as did other Joiners.

## Discussion

This analysis reads institution's reports as holding implicit estimates of a trend in frequency of restrained people per month per monthly bed-days, as plotted on a log-log graph to handle effects of scale.

Reporting remains incomplete per the literature and publicly available documents. Our funnel and scatter plots add a suggestion omissions of restrain reports may not be random. We show that restraint seems related to size and people detained, which is a helpful addition to the discourse, and has face validity.

Janssen et al. 2011 aimed to correct for five factors in their institutional analysis namely regional size, urbanicity, secure care, beds and admissions. We are constrained by externally defined metrics and a manifestly incomplete dataset. In England, the largest institutions have secure wards. Our method does not correct directly for regional size or urbanicity, but calls upon bed days and people detained to be proxies for them, and benefits from people detained as a comparator which takes acuity into account.

Use of three reasonably independent metrics (restraint, size, people detained) allows some internal validation. Furthermore, we use that insight to show how restrained people per month might be estimated, in principle, among incompletely reporting institutions. That may be of use to regulators.

This analysis used funnel plots (7). Classically these visualise relationships between study precisions, and study estimates, of a presumed common effect. Various causes of asymmetric funnels exist. Essentially, a symmetrical funnel suggests a set of estimates is relatively unaffected by systemic errors, e.g. bias. An even funnel has precise estimates agreeing at the “top”, on a midline; and less precise (weaker, smaller, more pragmatically variable) estimates evenly and increasingly spread toward the “bottom”. The funnels for restraint by size and restraint by people detained were very asymmetrical. Our funnel plots suggest that less precise reports tend toward underestimation. It may reflect other effects than “pure” publication bias. It is difficult to conceive of true causative effects that would link imprecise reporting to lower restraint. However, we offer other speculative explanations.

Perhaps most imprecision in restraint reporting is caused by omissions; intuitively omissions of restraints are more likely than spurious “over-reporting” of fictive people restrained per month. Alternatively, institutions that restrain most might consequently develop precise measurement. Conversely, highly restraining institutions who report highly imprecise restraint rates may close or be subsumed.

Inescapably, though, readers may postulate publication bias. Null reports may be voluntary. Institutions might “stay out” of reporting fearing unfair comparison, or for other reasons. New joiners are precise, and consistent, once they join. Perhaps they perfected reporting methods before sharing their data.

There are limitations to these methods. The first author's employment by a large and candid NHS trust may paradoxically invite concerns of bias. The underlying data may be rounded; but that is owned by NHS Digital, for good reasons. The data is incomplete; but the incompleteness is our point. Categorisations may be subjective; but categorisations would be unnecessary if everyone reported completely – all reporters would be in the complete category, which allowed 1 month omission.

There is numerical mismatch between size-based and “people detained”-based estimates among some null reporters, above a certain estimated level of restraint/size/people detained. It may be explained by those factors which interact with size, characteristics such as patient age, neurodiversity; or, more speculatively saturation of restraint and/or reporting, both of which depend on finite resources. We cannot know until reports are complete, per person, per month, along with ethnic and other features, as the law will require soon. Corrections such as those suggested by Janssen rely on complete data for the corrective factors, and we look to a day when we can make them in England.

The exclusion of an outlying null-reporting trust, helped our very high (0.95) correlation of size and people detained estimates. It required checking a null reporting outlier's CQC report *post hoc*. That retrospective check particularly looked for issues with MHA reporting. This may entail bias. That is our own main methodological concern.

If “people restrained” per month is imperfectly reported, “number of restraints” may be worse. Anecdotally, institutional definitions of restraint vary, especially for “prone”. Numerous varying exceptions are rumoured. We predict and recommend that regulators should acknowledge varying definitions, and ask patients (detained or not) if they have been people restrained in a month.

Seni Lewis whose death in restraint led to the Mental Health Units Use of Force Act had a Master's degree in Information Technology and Management. As a theoretical extension, the first author developed a measure “L” to quantify disinformation added by low or null reports using bits. It is named for the scatter graph shape and after “Lewis”, with the family's kind permission (8). As with PROD-ALERT the methods for L-test are intended to be open source.

## Data Availability

Publicly available datasets were analyzed in this study. This data can be found here: https://digital.nhs.uk/data-and-information/data-collections-and-data-sets/data-sets/mental-health-services-data-set.
